# Impact of divergence of residual feed intake on triglyceride metabolism-related gene expression in meat-type ducks

**DOI:** 10.1371/journal.pone.0286051

**Published:** 2023-05-22

**Authors:** Fei Shui, Guiru Qiu, Shenqiang Pan, Xin Wang, Tingting Jiang, Zhaoyu Geng, Sihua Jin

**Affiliations:** 1 College of Animal Science and Technology, Anhui Agricultural University, Hefei, China; 2 Anhui Provincial Key Laboratory of Local Animal Genetic Resources Conservation and Bio-Breeding, Hefei, China; Universidade Federal de Mato Grosso do Sul, BRAZIL

## Abstract

Triglyceride (TG) metabolism is a key factor that affects residual feed intake (RFI); however, few studies have been conducted on the related gene expression in poultry. The aim of the present study was to investigate the expression of genes and their associations with RFI in meat-type ducks. Weight gain and feed intake (FI) at an age 21–42 days were measured and the RFI was calculated. Quantitative PCR was used to test the expression of the six identified genes, namely peroxisome proliferator activated receptor γ (*PPARγ*), glycerol kinase 2 (*GK2*), glycerol-3-phosphate dehydrogenase 1 (*GPD1*), glycerol kinase (*GYK*), lipase E (*LIPE*), and lipoprotein lipase (*LPL*) in the duodenum in the high RFI (HRFI) and low RFI (LRFI) groups. The results demonstrated that daily feed intake, feed conversion ratio (FCR), and RFI were markedly higher in HRFI ducks than those in LRFI ducks. Moreover, the levels of expression of *PPARγ*, *GK2*, and *LIPE* were significantly higher in the LRFI group than those in the HRFI group. Correlation analysis showed that *PPARγ*, *GK2*, and *LIPE* were significantly negatively associated with FCR and RFI. Furthermore, gene expression levels were negatively associated with the measured phenotype. The association of *GK2* with *PPARγ*, *GPD1*, *LPL*, and *LIPE* was positive. The relationship between the TG related gene and RFI was further verified to potentially develop pedigree poultry breeding programs. The results of this study suggested that the expression of genes correlated with TG metabolism and transport is up-regulated in the duodenum of ducks with high feed efficiency. *PPARγ*, *GK2*, and *LIPE* are important genes that affect RFI. The results of the present study provide information that could facilitate further explorations of the mechanism of RFI and potential markers at the molecular and cellular levels.

## Introduction

The poultry industry in China is well developed and plays a pivotal role in the agricultural economy. As poultry, ducks receive the largest amount of feed in China and have been an important part of the livestock production. In modern breeding, feed cost accounts for approximately 70% of the total raising cost [1]. To increase the benefit of meat-type duck breeding, research is currently focusing on improving feed efficiency (FE).

FE is evaluated based on feed conversion ratio (FCR) and residual feed intake (RFI) [2]. Kennedy [3] proposed the RFI value, which is defined as the difference between the actual and expected feed intake (FI) based on the weight gain and average animal weight. As an evaluation index of FE for livestock and poultry, RFI is based on weight difference, independent of production [4], and has medium heritability [5]. RFI can be selected for application in FE improvement, whereas FCR is a ratio trait with limited breeding applications. Therefore, as RFI is a useful assessment for FE in meat-type duck breeding, researchers are committed to finding the potential genetic markers of RFI [1, 6].

Lipid metabolism is a key factor affecting RFI. Different levels of energy are consumed when depositing lean tissue and fat of an equivalent weight. The energy metabolism of low RFI (LRFI) animals is reflected in improvements in nutrition and energy distribution efficiency for growth and muscle deposition [7]. In poultry, LRFI broilers had lower abdominal fat rates and higher breast muscle rates than HRFI broilers [8], and both abdominal fat and breast muscle rates were strikingly correlated with the breeding value of RFI traits [9]. As a product of lipolysis, triglyceride (TG) decomposition and synthesis is an essential part of lipid metabolism. The total amount of fat deposition and types of fatty acids are the products of the comprehensive regulation of TG synthesis and metabolism in the body, which are regulated at different levels [10]. Based on previous studies, we selected six key genes involved in triglyceride synthesis and catabolism. *GYK* [11], *GK2* [12], and *GPD1* [13] are involved in the synthesis of TG, while *PPARγ* [14], *LIPE* [15], and *LPL* [16] are key genes involved in the catabolism of TG. The duodenum is an important site for the decomposition and utilization of TG [17]. The activity of pancreatic lipase in the intestine can affect the absorption of fat in the body [18]. However, few studies have compared the expression characteristics of genes related to TG metabolism in the duodenum of different meat-type ducks.

Therefore, in the present study, we evaluated the complete genealogical records of the Qiangying duck Breeding Co. Ltd., China using quantitative PCR to explore the TG metabolism-related gene expression differences in HRFI and LRFI and its relevance to RFI. We provide a theoretical basis for the improved breeding of meat-type ducks and selection of high FE ducks to reveal the control of the biological mechanisms associated with RFI.

## Materials and methods

### Ethics statement

All protocols including animal experiments were carried out in adherence to the regulations and guidelines established by the Administration of Affairs Concerning Experimental Animals (Ministry of Science and Technology, China, revised in June 2004). All experimental procedures were reviewed and approved by the Institutional Animal Care and Use Committee of Anhui Agricultural University (SYXK 2016–007). The animals in the study had *ad libitum* access to water and feed and were humanely euthanized by cervical dislocation. All efforts were made to relieve the suffering of the ducks.

### Birds, diets, and management

Healthy and strong Huangshan Qiangying ducks with complete pedigree records and clear provenance were selected as the experimental material, and were provided by Huangshan Qiangying Duck Breeding Co. Ltd. (Anhui, China). A total of 1100 one-day-old healthy male ducks with similar body weight (60.1 ± 2.3 g) were selected for rearing in the same duck house until 21 days of age. At 21 days, all ducks were weighed, and a total of 1000 meat-type male ducks with similar body weight (1042.1 ± 87.2 g) were placed in the individual cages (55 × 50 × 40 cm) for feeding until 42 days of age. All ducks were exposed to continuous illumination (24 L: 0 D) for the first three days after hatching, followed by a 20 L: 4 D lighting regime until the end of the experiment. All ducks were housed in the same house and fed the same basal diet at room temperature. During the experiment, the feeding management and immunization procedures were carried out according to uniform standards, and all the experimental ducks were free to feed and water *ad libitum*. Ingredient composition and nutrient levels of the basal diet used in the present study are shown in Table 1.

**Table 1:** Ingredient and chemical composition of feed from hatch to 21 days and till 42 days (% as fed).

Item	Nutritional ratio
**Composition (%)**	
Corn	59.22
Wheat bran	11.94
Soybean meal	25.32
Limestone	1.20
Dicalcium phosphate	1.40
Sodium chloride	0.30
DL-Methionine	0.12
Vitamin and trace mineral premix	0.50
Total	100.0
**Nutrient levels**	
Metabolizable energy, kcal/kg	2950
Crude protein(%)	17.50
Methionine(%)	0.40
Cystine(%)	0.32
Lysine(%)	1.05
Calcium(%)	0.86
Non-phytate phosphorus(%)	0.38

Supplied the following per kilogram of total diet: Cu (CuSO_4_·5H_2_O), 10 mg; Fe (FeSO_4_ 7H_2_O), 60 mg; Zn (ZnO), 60 mg; Mn (MnSO_4_·H_2_O), 80 mg; Se (NaSeO_3_), 0.3 mg; I (KI), 0.2 mg; Cr (Cr_2_O_3_), 0.15 mg; choline chloride, 750 mg; vitamin A (retinyl acetate), 8,000 IU; vitamin D3 (cholecalciferol), 3,000 IU; vitamin E (DL-α-tocopheryl acetate), 20 IU; vitamin K3 (menadione sodium bisulfate), 2 mg; thiamin(thiamin mononitrate), 1.5 mg; riboflavin, 8 mg; pyridoxine hydrochloride, 3 mg; cobalamin, 0.02 mg; calcium-D-pantothenate, 10 mg; nicotinic acid, 50 mg; folic acid, 1 mg; biotin, 0.2 mg.

### Trait measurement

We recorded the 21-day-old body weight (BW21), 42-day-old body weight (BW42), FI, body weight gain (BWG), and calculated the average daily feed intake (ADFI) and average daily gain (ADG), FCR, and RFI [19, 20], using the following formula of RFI:

$$RFI=ADFI-(b_{0}+b_{1}\times MBW^{0.75}+b_{2}\times ADG)$$

where RFI is the residual feed intake; ADFI is the daily feed intake; MBW^0.75^ is the metabolic body weight; ADG is the average daily gain; *b*_0_ is the intercept, and *b*_1_ and *b*_2_ are regression coefficients. The linear fitting function in SAS version 9.4 software (SAS Institute, Cary, NC, USA) was used to calculate the RFI value of the meat-type ducks.

### Sample collection and total RNA extraction

At 42 days of age, the RFI of all individuals was determined. According to the results of the RFI ranking, the experimental duck population was divided into HRFI and LRFI groups, and considering their FCR values, eight meat-type male ducks were selected each group. After the ducks were slaughtered, the duodenum was collected and immediately stored in a 2.0 mL RNA containing centrifuge tube at 4°C overnight and then stored at −80°C until further analyses. Total RNA from duodenal epithelial tissues was extracted using a total RNA kit (Omega Bio-Tek, Doraville, GA, USA). The RNA concentration and purity of samples were determined using NanoDrop spectrophotometer (Thermo Fisher Scientific, New York, NY, USA) in 1.0% agarose gel electrophoresis. SuperMix was synthesized using cDNA for qPCR (Yeasen, Shanghai, China) for the reverse transcription of the isolated total RNA. All experimental procedures were carried out as per the manufacturer’s protocols.

### Primers, complementary DNA synthesis, and quantitative PCR analysis

According to the duck genome sequence published in the GenBank, the primers of six genes were designed using Primer 5.0 software (Premier Biosoft International, Palo Alto, CA, USA), as shown in Table 2. Hydroxymethylbilane synthase (*HMBS*) was employed as an internal reference gene, and quantitative PCR was performed using the Hieff qPCR SYBR Green Master Mix kit (Yeasen, Shanghai, China) with the ABI 7500 Fast Real-Time PCR System (Applied Biosystems, Carlsbad, CA, USA) according to manufacturer’s instructions. Thermal cycling parameters were as follows: 95°C for 5 min, followed by 40 cycles of 10 s at 95°C, 20 s at 60°C, and 20 s at 72°C, and a final step of 5 min at 72°C. The reaction specificity was confirmed by melt curve analysis. The relative transcriptional levels of the various genes were confirmed using the 2^-ΔΔCt^ (in which Ct is the cycle threshold period) method.

**Table 2:** Specific primers used for quantitative PCR.

Gene^1^	Accession no.	Primer^2^ (5’ to 3’)	Length (bp)	Annealing temperature (°C)
*PPARγ*	XM_027467224.1	F:ACCACAGATCAACCCAGAGG	187	60
		R:TCAGATGTTTCCCAGCCCAT		
*GK2*	XM_003640510.4	F:ACCACAGATCAACCCAGAGG	87	60
		R:TCAGATGTTTCCCAGCCCAT		
*GPD1*	XM_005021562.4	F:TGAAGGAGCTGATGCAGACA	96	60
		R:CAGCCACGATGTTCTTCAGG		
*GYK*	XM_002295867.1	F:TTCTGATGGATCGTCGTCGT	120	60
		R:ATATCCAAGCCACGTTGTGC		
*LIPE*	XM_005258937.3	F:AGGCTCATCCACAACATGGA	87	60
		R:CTGGCTCGAGAAGAAGGCTA		
*LPL*	XM_027446391.1	F:TGTCTGCTACCTGGTTCCTG	64	60
		R:ACTGGTGTGGTTGAAGTTGC		
*HMBS*	XM_027444343	F: TGGACCAAATGACGATGTGC	174	60
		R: CCACAGGTTTAGCAGGCATC		

^1^peroxisome proliferator activated receptor gamma (*PPARγ*), glycerol kinase 2 (*GK2*), glycerol-3-phosphate dehydrogenase 1 (*GPD1*), glycerol kinase (*GYK*), lipase E (*LIPE*), lipoprotein lipase (*LPL*), hydroxymethylbilane synthase (*HMBS*).

^2^F = forward, R = reverse.

### Statistical analysis

The 2^−ΔΔCt^ method was used to calculate the relative gene expression levels according to the Ct values of the target and internal genes [21]. Experiments were based on using a completely randomized design, and data were analyzed using the general linear model procedure of IBM SPSS Statistics 25 (IBM Corp., Armonk, NY, USA). Differences in phenotypic data and relative expression of TG metabolizing-related genes between the HRFI and LRFI groups were analyzed by students′ *t*-test in IBM SPSS Statistics 25. Spearman correlation coefficients among feed efficiency traits, growth traits, and gene expression values were calculated using the PROC CORR command in IBM SPSS Statistics 25. Data are expressed as mean ± standard deviation. A *P* < 0.05 indicates that the difference is statistically significant.

## Results

### Feed efficiency traits

FE trait data are presented in Table 3, showing that ADFI, FCR, and RFI were significantly lower (*P* < 0.01) in LRFI ducks than those in HRFI ducks. However, there was no significant difference in ADG and MBW^0.75^ between the HRFI and LRFI groups (*P* > 0.05).

**Table 3:** Comparison of residual feed intake and relevant traits between high and low residual feed intake (HRFI and LRFI) ducks.

Trait^1^	HRFI (n = 8)	LRFI (n = 8)
ADFI, g/d	301.67±13.20^A^	252.45±12.37^B^
ADG, g/d	133.60±7.58	134.10±6.51
MBW^0.75^, g/d	359.32±13.96	350.68±15.29
FCR, g/g	2.26±0.05^A^	1.88±0.02^B^
RFI, g/d	24.42±2.16^A^	-19.90±2.08^B^

^1^ADFI: average daily feed intake; ADG: average daily gain; MBW^0.75^: metabolic body weight; FCR: feed conversion ratio; RFI: residual feed intake.

Different uppercase letters in the same line means differ significantly (*P* < 0.01).

### Gene expression

**Fig 1:**
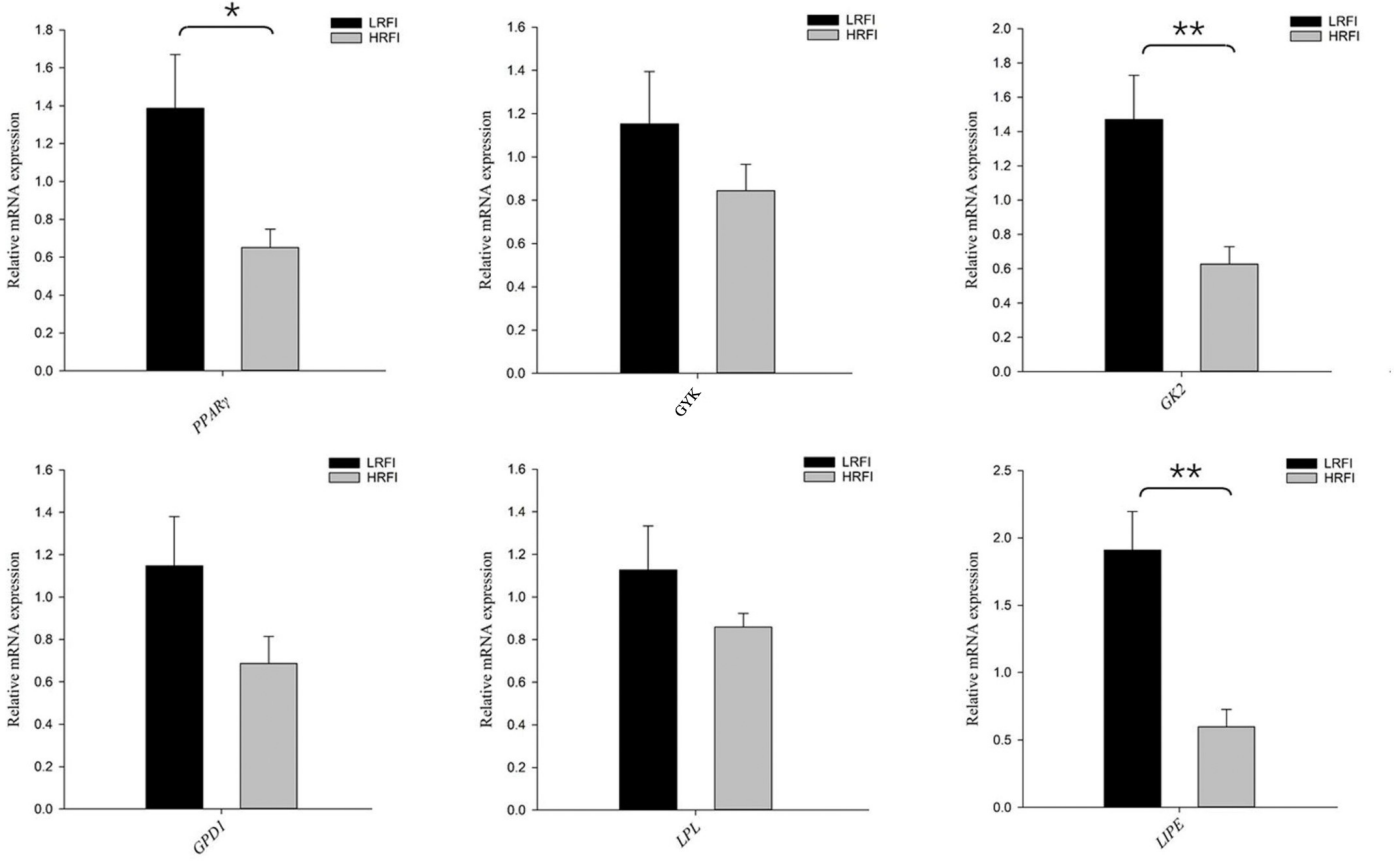
Gene expression analysis of high and low residual feed intake (HRFI and LRFI) groups. Peroxisome proliferator activated receptor gamma (*PPARγ*), glycerol kinase (*GYK*), glycerol kinase 2 (*GK2*), glycerol-3-phosphate dehydrogenase 1 (*GPD1*), lipoprotein lipase (*LPL*), lipase E (*LIPE*). * = *P* < 0.05; ** = *P* < 0.01.

The relative levels of expression of TG metabolism-related genes in the duodenums of HRFI and LRFI groups are displayed in Fig 1. The expression levels of *PPARγ* (*P* < 0.05), *GK2* (*P* < 0.01), and *LIPE* (*P* < 0.01) in the LRFI group were markedly higher than those in the HRFI group. There was no statistical difference in *GYK*, *GPD1*, and *LPL* between the two RFI groups (*P* > 0.05).

### Relationship between gene expression and feed efficiency traits

As shown in Table 4, the relative expression levels of *PPARγ* were significantly and negatively correlated with MBW^0.75^ (*P* < 0.05), FCR (*P* < 0.05), RFI (*P* < 0.05), and ADFI (*P* < 0.01). Similarly, the relative levels of expression of *GK2* were negatively correlated with MBW^0.75^ (*P* < 0.05), RFI (*P* < 0.05), ADFI (*P* < 0.01), and FCR (*P* < 0.01). The relative levels of expression of *LIPE* were significantly negatively correlated with ADFI, FCR, and RFI (*P* < 0.01). It can be concluded that the relative levels of expression of *PPARγ*, *GK2*, and *LIPE* were negatively correlated with ADFI, FCR, and RFI in the meat-type ducks.

**Table 4:** Associations between the expression levels of genes related to triglyceride metabolism in the duodenum and phenotypic traits in meat-type ducks^1^^,^^2^.

Gene^3^	ADFI	ADG	MBW^0.75^	FCR	RFI
*PPARγ*	-0.6601**	-0.3046	-0.5886*	-0.5518*	-0.5101*
*GYK*	-0.2481	0.0069	-0.0743	-0.2670	-0.2774
*GK2*	-0.6945**	-0.2254	-0.5131*	-0.6368**	-0.6021*
*GPD1*	-0.4070	0.0212	-0.2477	-0.4556	-0.4088
*LPL*	-0.4023	-0.2414	-0.4202	-0.3051	-0.2743
*LIPE*	-0.6661**	0.0567	-0.3188	-0.7611**	-0.7082**

^1^ADFI: average daily feed intake; ADG: average daily gain; MBW: metabolic body weight; FCR: feed conversion ratio; RFI: residual feed intake.

^2^n = 8 (HRFI); n = 8 (LRFI).

^3^peroxisome proliferator activated receptor gamma (*PPARγ*), glycerol kinase (*GYK*), glycerol kinase 2 (*GK2*), glycerol-3-phosphate dehydrogenase 1 (*GPD1*), lipoprotein lipase (*LPL*), lipase E (*LIPE*).

* = *P* < 0.05

** = *P* < 0.01.

### Correlation between gene expression and triglyceride metabolism

The results of the correlation analysis based on relative gene expression are shown in Table 5. The expression of TG metabolism-related genes showed a positive correlation, of which, *PPARγ* gene expression was significantly positively correlated with the expression of *GK2*, *GPD1*, *LPL*, and *LIPE* (*P* < 0.01). *GYK* expression was positively correlated with *GPD1* expression (*P* < 0.05). *GK2* expression was significantly positively correlated with *GPD1*, *LPL*, and *LIPE* expression (*P* < 0.01). *GPD1* expression was significantly positively correlated with *LPL* and *LIPE* expression (*P* < 0.01). *LPL* expression was positively correlated with *LIPE* expression (*P* < 0.05).

**Table 5:** Correlation between gene expression and triglyceride metabolism in the duodenum of meat-type ducks^1^.

Gene^2^	*PPARγ*	*GYK*	*GK2*	*GPD1*	*LPL*	*LIPE*
*PPARγ*	1	0.3533	0.8402**	0.6842**	0.8401**	0.6908**
*GYK*		1	0.4913	0.5714*	0.4277	0.3953
*GK2*			1	0.6722**	0.7551**	0.7561**
*GPD1*				1	0.7165**	0.7204**
*LPL*					1	0.5312*
*LIPE*						1

^1^n = 8 (HRFI); n = 8 (LRFI).

^2^peroxisome proliferator activated receptor gamma (*PPARγ*), glycerol kinase (*GYK*), glycerol kinase 2 (*GK2*), glycerol-3-phosphate dehydrogenase 1 (*GPD1*), lipoprotein lipase (*LPL*), lipase E (*LIPE*).

* = *P* < 0.05

** = *P* < 0.01.

## Discussion

Enhancing the FE of livestock and poultry could reduce breeding costs, increase the economic benefits of breeding, and reduce environmental pollution [22]. In the present study, the ADFI, FCR, and RFI of meat-type ducks were remarkably different between HRFI and LRFI groups (*P* < 0.01). The results are in agreement with the findings of previous studies on chickens [8] and mule ducks [23]. These studies indicate that poultry with LRFI has higher FE and lower breeding costs. Zhang et al. [1] reported that the sebum rate of Peking ducks was remarkably positively correlated with RFI. Individuals with LRFI have lower fat content and lower expression of various proteins related to fat deposition synthesis such as fatty acid synthase, fatty acid binding protein, and peripherin. Zhuo et al. [8] found that chicken abdominal fat that differentially expressed genes were mainly enriched in lipid metabolism, coagulation, and immune regulatory pathways.

The duodenum is an important organ for nutrient digestion and absorption. Most of the fat in the duodenum is absorbed into intestinal cells by enzymes, and then synthesized and stored in the body. Feed intake is an important factor affecting RFI [7], and dietary fat is mostly consumed in the form of TG. Previous transcriptome studies of the duodenum of chicken showed that RFI was related to the regulation of nutrient digestion and absorption and that intestinal health can improve FE. Ten genes related to digestive health and function were also identified as candidate genes for RFI [6].

Glycerol metabolism has a major effect on the catabolism of fatty acids and sugars [24]. The *PPAR* signaling pathway plays crucial roles in lipid metabolism, adipogenesis, and insulin sensitivity. *PPARγ* belongs to the nuclear receptor superfamily of ligand-activated transcription factors (TFs) [25, 26]. It is highly expressed in adipocytes and is a key regulator of adipocyte differentiation, insulin signaling, fat energy conversion, bone morphogenesis and differentiation, and immune response [27–29]. It plays a pivotal role in lipid metabolism and related diseases, such as obesity [30], diabetes [31], atherosclerosis [32], and cancer [33]. *PPARγ* can control lipid metabolism and monounsaturated fatty acid synthesis [14] and regulate TG synthesis in mammary epithelial cells of goats [34] and cows [35]. Increased *PPARγ* expression in adipose tissue has also been observed in low FE mice [36]. Moreover, long-term feeding with the *PPARγ* activator activates *PPARγ* and improves FI and FE in rats, accompanied by high leptin levels, when compared to rats in other high-fat feeding groups [37]. Chicken *PPARγ* is co-expressed in adipocytes with other adipogenesis-related TFs and regulates the expression of adipogenesis-related genes [38]. However, the expression of *PPARγ* in the duodenum is different from that in adipose tissue. Ojano-Dirain et al. [39] found that *PPARγ* expression in the duodenum of chickens with high FE was higher than that of chickens with low FE, which is consistent with the results of our study. At the same time, Kelly et al. [40] found that *PPARγ* expression was negatively correlated with FCR and RFI, which was also consistent with the results of our study. This may be because, during fat accumulation, *PPARγ* can also promote TG to release fatty acids, ensure intercellular fatty acid transport, and promote fatty acid esterification by activating fatty acid binding proteins and lipoprotein esterase [26]. Since avian lipogenesis mainly occurs in the liver, and the duodenum is the place for nutrient digestion and absorption, the expression of *PPARγ* in the duodenum of HRFI meat-type ducks is distinctly lower than that of LRFI meat-type ducks. This study found that the expression level of *PPARγ* was negatively associated with ADFI, MBW^0.75^, FCR, and RFI, and the expression level of *PPARγ* in the duodenum of LRFI ducks was distinctly higher than that of HRFI. The phenotypic results were consistent with the differential expression of *PPARγ* in LRFI and HRFI. Upstream regions of *LPL* and fatty acid binding protein 4 (*FABP4*) promoters can bind to *PPAR*, after which *LPL* and *FABP4* are activated and expressed [41], which is consistent with the significant positive relationship between *PPARγ* and *LPL* expression levels observed in this study.

LIPE is an important member of the TG lipase family that mediates adipocyte lipolysis [42]. It is one of the key enzymes affecting fat deposition in animals. Greenberg et al. [43] first found this protein in epididymal adipocytes of mice. LIPE, as the rate limiting enzyme of TG hydrolysis, is responsible for converting TG into diacylglycerol [44, 45]. At the same time, the activity of this enzyme is controlled by hormones, which is also known as hormone sensitive lipase (HSL). Moreover, LIPE can not only catalyze the release of fatty acids from TG stored in adipocytes [15], but also catalyze the release of cholesterol from cholesterol esters in steroid tissues for steroid hormone synthesis [46]. The expression level of *LIPE* affects marbling and intramuscular fat (IMF) content, which is a potential molecular marker that affects bovine IMF, and it can be applied in breeding selection [45]. Liu et al. [47] used transcriptome analysis of differential expression of lipid metabolism-related genes in Shandong black and Luxi cattle and found that the *LIPE* is the key node of various metabolic pathways, and its relative expression is higher in cattle breeds with higher IMF content. The difference in the expression of *LIPE* may augment fatty acid related metabolic pathways of beef cattle and positively mediate polyunsaturated fatty acids, stearic acids, and linoleic acids. In a study of lipid metabolism in piglets, when low density lipoprotein (LDL) and cholesterol levels in the blood decreased and high density lipoprotein (HDL) increased, the expression of *LIPE* and *LPL* decreased significantly [48], in agreement with the gene expression observed in the present study. In our study, the expression of *LIPE* in meat-type ducks in the HRFI group was markedly higher than that in the LRFI group (*P* < 0.01), and *LIPE* was markedly positively associated with ADFI and FCR, which may be because LIPE is the rate limiting enzyme for TG hydrolysis. In addition, there was a positive relationship between the gene expression of *LIPE* and that of *LPL*.

GYK and GK2 are the enzymes responsible for converting glycerol into glycerol 3-phosphate (G3P), the substrate for glycolysis and lipid synthesis [11, 12, 24], as glycerol levels are elevated in the absence of GK [49]. Considering that glycerol levels usually reflect the state of fat mobilization, Assis et al. [50] found that glucocorticoids decrease thermogenic capacity and increase TG synthesis by GK activation in the brown adipose tissue of rats. In our study, *GK2* was negatively correlated with MBW^0.75^, RFI, ADFI, and FCR. In addition, the level of expression of *GK2* in the LRFI group was markedly higher than that in the HRFI group. The change in the gene expression level may affect the TG metabolism of HRFI and LRFI meat-type ducks, and then in turn affect the lipid metabolism process of the body, changing the RFI.

## Conclusion

In conclusion, our findings indicated that the expression of *PPARγ*, *GK2*, and *LIPE* involved in TG metabolism and transport was significantly up-regulated in the duodenum of meat-type ducks with high feed efficiency. Moreover, association analysis indicated that the relative expression levels of *PPARγ*, *GK2*, and *LIPE* were significantly and negatively correlated with FCR, RFI, and ADFI. Additionally, there was a significant positive correlation between the relative expression of *PPARγ*, *GK2*, and *LIPE* genes.

## References

[1] Zhang Y, Guo ZB, Xie M, Zhang Z, Hou S. Genetic parameters for residual feed intake in a random population of Pekin duck. Asian-Australas J Anim Sci. 2017; 30(2):167–170. doi: 10.5713/ajas.15.0577 . 27165030 PMC5205602

[2] Case LA, Wood BJ, Miller SP. The genetic parameters of feed efficiency and its component traits in the turkey (Meleagris gallopavo). Genet Sel Evol. 2012; 44(1):2. doi: 10.1186/1297-9686-44-2 . 22268922 PMC3296663

[3] Kennedy BW, van der Werf JH, Meuwissen TH. Genetic and statistical properties of residual feed intake. J Anim Sci. 1993; 71(12):3239–3250. doi: 10.2527/1993.71123239x . 8294275

[4] Davis ME, Simmen RCM. Genetic parameter estimates for serum insulin-like growth factor I concentrations, and body weight and weight gains in Angus beef cattle divergently selected for serum insulin-like growth factor I concentration. J Anim Sci. 2006; 84(9):2299–2308. doi: 10.2527/jas.2005-567 . 16908632

[5] Gilbert H, Bidanel JP, Gruand J, Caritez JC, Billon Y, Guillouet P, et al. Genetic parameters for residual feed intake in growing pigs, with emphasis on genetic relationships with carcass and meat quality traits. J Anim Sci. 2007; 85(12):3182–3188. doi: 10.2527/jas.2006-590 . 17785600

[6] Liu R, Liu J, Zhao G, Li W, Zheng M, Wang J, et al. Relevance of the intestinal health-related pathways to broiler residual feed intake revealed by duodenal transcriptome profiling. Poult Sci. 2019; 98(3):1102–1110. doi: 10.3382/ps/pey506 . 30452726

[7] Herd RM, Arthur PF. Physiological basis for residual feed intake. J Anim Sci. 2009; 87(14 Suppl):E64–71. doi: 10.2527/jas.2008-1345 . 19028857

[8] Zhuo Z, Lamont SJ, Lee WR, Abasht B. RNA-Seq analysis of abdominal fat reveals differences between modern commercial broiler chickens with high and low feed efficiencies. PLoS One. 2015; 10(8):e0135810. doi: 10.1371/journal.pone.0135810 . 26295149 PMC4546421

[9] Hakimeh, Rasoul Ali, Alireza E, Just J. Relationship between residual feed intake and carcass composition, meat quality and size of small intestine in a population of F2 chickens. Livest Sci. 2017; 205:10–15. 10.1016/j.livsci.2017.09.001.

[10] Alves-Bezerra M, Cohen DE. Triglyceride Metabolism in the Liver. Compr Physiol. 2017; 8(1):1–8. doi: 10.1002/cphy.c170012 . 29357123 PMC6376873

[11] Miao L, Yang Y, Liu Y, Lai L, Wang L, Zhan Y, et al. Glycerol kinase interacts with nuclear receptor NR4A1 and regulates glucose metabolism in the liver. FASEB J. 2019; 33(6):6736–6747. doi: 10.1096/fj.201800945RR . 30821173

[12] Shimada K, Kato H, Miyata H, Ikawa M. Glycerol kinase 2 is essential for proper arrangement of crescent-like mitochondria to form the mitochondrial sheath during mouse spermatogenesis. J Reprod Dev. 2019; 65(2):155–162. doi: 10.1262/jrd.2018-136 . 30662012 PMC6473107

[13] Wu JW, Yang H, Wang SP, Soni KG, Brunel-Guitton C, Mitchell GA. Inborn errors of cytoplasmic triglyceride metabolism. J Inherit Metab Dis. 2015; 38(1):85–98. doi: 10.1007/s10545-014-9767-7 . 25300978

[14] Shi HB, Luo J, Yao DW, Zhu JJ, Xu HF, Shi HP, et al. Peroxisome proliferator-activated receptor-gamma stimulates the synthesis of monounsaturated fatty acids in dairy goat mammary epithelial cells via the control of stearoyl-coenzyme A desaturase. J Dairy Sci. 2013; 96(12):7844–7853. doi: 10.3168/jds.2013-7105 . 24119817

[15] Zhang X, Zhang CC, Yang H, Soni KG, Wang SP, Mitchell GA, et al. An Epistatic Interaction between Pnpla2 and Lipe Reveals New Pathways of Adipose Tissue Lipolysis. Cells. 2019; 8(5):395. doi: 10.3390/cells8050395 . 31035700 PMC6563012

[16] Shi H, Wang Z. Novel pathogenic variant combination in LPL causing familial chylomicronemia syndrome in an Asian family and experimental validation in vitro: a case report. Transl Pediatr. 2022; 11(10):1717–1725. doi: 10.21037/tp-22-15 . 36345447 PMC9636460

[17] Ko CW, Qu J, Black DD, Tso P. Regulation of intestinal lipid metabolism: current concepts and relevance to disease. Nat Rev Gastroenterol Hepatol. 2020; 17(3):169–183. doi: 10.1038/s41575-019-0250-7 . 32015520

[18] Liu TT, Liu XT, Chen QX, Shi Y. Lipase inhibitors for obesity: A review. Biomed Pharmacother. 2020; 128:110314. doi: 10.1016/j.biopha.2020.110314 . 32485574

[19] Jin S, Xu Y, Zang H, Yang L, Lin Z, Li Y, et al. Expression of genes related to lipid transport in meat-type ducks divergent for low or high residual feed intake. Asian-Australas J Anim Sci. 2020; 33(3):416–423. doi: 10.5713/ajas.19.0284 . 31480135 PMC7054623

[20] Aggrey SE, Karnuah AB, Sebastian B, Anthony NB. Genetic properties of feed efficiency parameters in meat-type chickens. Genet Sel Evol. 2010; 42(1):25. doi: 10.1186/1297-9686-42-25 . 20584334 PMC2901204

[21] Livak KJ, Schmittgen TD. Analysis of relative gene expression data using real-time quantitative PCR and the 2(-Delta Delta C(T)) Method. Methods. 2001; 25(4):402–408. doi: 10.1006/meth.2001.1262 . 11846609

[22] Saintilan R, Merour I, Brossard L, Tribout T, Dourmad JY, Sellier P, et al. Genetics of residual feed intake in growing pigs: Relationships with production traits, and nitrogen and phosphorus excretion traits. J Anim Sci. 2013; 91(6):2542–2554. doi: 10.2527/jas.2012-5687 . 23482579

[23] Drouilhet L, Monteville R, Molette C, Lague M, Cornuez A, Canario L, et al. Impact of selection for residual feed intake on production traits and behavior of mule ducks. Poult Sci. 2016; 95(9):1999–2010. doi: 10.3382/ps/pew185 . 27333975 PMC4983686

[24] Yeh JI, Kettering R, Saxl R, Bourand A, Darbon E, Joly N, et al. Structural characterizations of glycerol kinase: unraveling phosphorylation-induced long-range activation. Biochemistry. 2009; 48(2):346–356. doi: 10.1021/bi8009407 . 19102629 PMC3158585

[25] Evans RM, Barish GD, Wang YX. PPARs and the complex journey to obesity. Nat Med. 2004; 10(4):355–361. doi: 10.1038/nm1025 . 15057233

[26] Rosen ED, Spiegelman BM. PPARgamma: a nuclear regulator of metabolism, differentiation, and cell growth. J Biol Chem. 2001; 276(41):37731–37734. doi: 10.1074/jbc.R100034200 . 11459852

[27] Lecka-Czernik B. Bone loss in diabetes: use of antidiabetic thiazolidinediones and secondary osteoporosis. Curr Osteoporos Rep. 2010; 8(4):178–184. doi: 10.1007/s11914-010-0027-y . 20809203 PMC2947013

[28] Arner P. The adipocyte in insulin resistance: key molecules and the impact of the thiazolidinediones. Trends Endocrinol Metab. 2003; 14(3):137–145. doi: 10.1016/s1043-2760(03)00024-9 . 12670740

[29] Lee CH, Olson P, Evans RM. Minireview: lipid metabolism, metabolic diseases, and peroxisome proliferator-activated receptors. Endocrinology. 2003; 144(6):2201–2207. doi: 10.1210/en.2003-0288 . 12746275

[30] Shao X, Wang M, Wei X, Deng S, Fu N, Peng Q, et al. Peroxisome proliferator-activated receptor-gamma: Master regulator of adipogenesis and obesity. Curr Stem Cell Res Ther. 2016; 11(3):282–289. doi: 10.2174/1574888x10666150528144905 . 26018229

[31] Wang Q, Imam MU, Yida Z, Wang F. Peroxisome proliferator-activated receptor gamma (PPARgamma) as a target for concurrent management of diabetes and obesity-related cancer. Curr Pharm Des. 2017; 23(25):3677–3688. doi: 10.2174/1381612823666170704125104 . 28677503

[32] Zhang T, Shao B, Liu GA. Rosuvastatin promotes the differentiation of peripheral blood monocytes into M2 macrophages in patients with atherosclerosis by activating PPAR-gamma. Eur Rev Med Pharmacol Sci. 2017; 21(19):4464–4471. . 29077145

[33] Ramer R, Heinemann K, Merkord J, Rohde H, Salamon A, Linnebacher M, et al. COX-2 and PPAR-gamma confer cannabidiol-induced apoptosis of human lung cancer cells. Mol Cancer Ther. 2013; 12(1):69–82. 10.1158/1535-7163.MCT-12-0335 . 23220503

[34] Shi H, Zhao W, Zhang C, Shahzad K, Luo J, Loor JJ. Transcriptome-Wide analysis reveals the role of PPARgamma controlling the lipid metabolism in goat mammary epithelial cells. PPAR Res. 2016; 2016:9195680. doi: 10.1155/2016/9195680 . 27818678 PMC5081438

[35] Zhang MQ, Gao JL, Liao XD, Huang TH, Zhang MN, Wang MQ, et al. miR-454 regulates triglyceride synthesis in bovine mammary epithelial cells by targeting PPAR-gamma. Gene. 2019; 691:1–7. doi: 10.1016/j.gene.2018.12.048 . 30599237

[36] Nisoli E, Clementi E, Paolucci C, Cozzi V, Tonello C, Sciorati C, et al. Mitochondrial biogenesis in mammals: the role of endogenous nitric oxide. Science. 2003; 299(5608):896–899. doi: 10.1126/science.1079368 . 12574632

[37] Larsen PJ, Jensen PB, Sørensen RV, Larsen LK, Vrang N, Wulff EM, et al. Differential influences of peroxisome proliferator–activated receptorsγ and -α on food intake and energy homeostasis. Diabetes. 2003; 52(9):2249–2259. 10.2337/diabetes.52.9.2249. 12941763

[38] Bai P, Houten SM, Huber A, Schreiber V, Watanabe M, Kiss B, et al. Peroxisome proliferator-activated receptor (PPAR)-2 controls adipocyte differentiation and adipose tissue function through the regulation of the activity of the retinoid X receptor/PPARγ heterodimer. J Biol Chem. 2007; 282(52):37738–37746. doi: 10.1074/jbc.M701021200 . 17951580

[39] Ojano-Dirain C, Toyomizu M, Wing T, Cooper M, Bottje WG. Gene expression in breast muscle and duodenum from low and high feed efficient broilers. Poult Sci. 2007; 86(2):372–381. doi: 10.1093/ps/86.2.372 . 17234853

[40] Kelly AK, Waters SM, McGee M, Fonseca RG, Carberry C, Kenny DA. mRNA expression of genes regulating oxidative phosphorylation in the muscle of beef cattle divergently ranked on residual feed intake. Physiol Genomics. 2011; 43(1):12–23. doi: 10.1152/physiolgenomics.00213.2009 . 20923863

[41] Nakachi Y, Yagi K, Nikaido I, Bono H, Tonouchi M, Schonbach C, et al. Identification of novel PPARgamma target genes by integrated analysis of ChIP-on-chip and microarray expression data during adipocyte differentiation. Biochem Biophys Res Commun. 2008; 372(2):362–366. doi: 10.1016/j.bbrc.2008.05.037 . 18489901

[42] Nagashima S, Yagyu H, Takahashi N, Kurashina T, Takahashi M, Tsuchita T, et al. Depot-specific expression of lipolytic genes in human adipose tissues—association among CES1 expression, triglyceride lipase activity and adiposity. J Atheroscler Thromb. 2011; 18(3):190–199. doi: 10.5551/jat.6478 . 21081832

[43] Greenberg AS, Egan JJ, Wek SA, Garty NB, Blanchette-Mackie EJ, Londos C. Perilipin, a major hormonally regulated adipocyte-specific phosphoprotein associated with the periphery of lipid storage droplets. J Biol Chem. 1991; 266(17):11341–11346. 10.1016/S0021-9258(18)99168-4 . 2040638

[44] Haemmerle G, Zimmermann R, Zechner R. Letting lipids go: hormone-sensitive lipase. Curr Opin Lipidol. 2003; 14(3):289–297. doi: 10.1097/00041433-200306000-00009 . 12840660

[45] Kazala EC, Petrak JL, Lozeman FJ, Mir PS, Laroche A, Deng JT, et al. Hormone-sensitive lipase activity in relation to fat content of muscle in Wagyu hybrid cattle. Livest Prod Sci. 2003; 79(1):87–96. 10.1016/S0301-6226(02)00141-0.

[46] Recazens E, Mouisel E, Langin D. Hormone-sensitive lipase: sixty years later. Prog Lipid Res. 2021; 82:101084. doi: 10.1016/j.plipres.2020.101084 . 33387571

[47] Liu R, Liu X, Bai X, Xiao C, Dong Y. Different expression of lipid metabolism-related genes in Shandong black cattle and Luxi cattle based on transcriptome analysis. Sci Rep. 2020; 10(1):21915. doi: 10.1038/s41598-020-79086-4 . 33318614 PMC7736358

[48] Wu T, Li K, Lyu Y, Yi D, Zhao D, Wang L, et al. Trilactic glyceride regulates lipid metabolism and improves gut function in piglets. Front Biosci (Landmark Ed). 2020; 25(7):1324–1336. doi: 10.2741/4858 . 32114435

[49] Sjarif DR, Hellerud C, van Amstel JK, Kleijer WJ, Sperl W, Lacombe D, et al. Glycerol kinase deficiency: residual activity explained by reduced transcription and enzyme conformation. Eur J Hum Genet. 2004; 12(6):424–432. doi: 10.1038/sj.ejhg.5201172 . 15026783

[50] Assis AP, Silva KE, Lautherbach N, Morgan HJN, Garofalo MAR, Zanon NM, et al. Glucocorticoids decrease thermogenic capacity and increase triacylglycerol synthesis by glycerokinase activation in the brown adipose tissue of rats. Lipids. 2022; 57(6):313–325. doi: 10.1002/lipd.12358 . 36098349

